# Population Structure of *Peronospora effusa* in the Southwestern United States

**DOI:** 10.1371/journal.pone.0148385

**Published:** 2016-02-01

**Authors:** Rebecca Lyon, James Correll, Chunda Feng, Burt Bluhm, Sandesh Shrestha, Ainong Shi, Kurt Lamour

**Affiliations:** 1 Department of Entomology & Plant Pathology, University of Tennessee, Knoxville, Tennessee, United States of America; 2 Department of Plant Pathology, University of Arkansas, Fayetteville, Arkansas, United States of America; 3 Department of Horticulture, University of Arkansas, Fayetteville, Arkansas, United States of America; Leibniz-Institute of Vegetable and Ornamental Crops, GERMANY

## Abstract

*Peronospora effusa* is an obligate pathogen that causes downy mildew on spinach and is considered the most economically important disease of spinach. The objective of the current research was to assess genetic diversity of known historical races and isolates collected in 2014 from production fields in Yuma, Arizona and Salinas Valley, California. Candidate neutral single nucleotide polymorphisms (SNPs) were identified by comparing sequence data from reference isolates of known races of the pathogen collected in 2009 and 2010. Genotypes were assessed using targeted sequencing on genomic DNA extracted directly from infected plant tissue. Genotyping 26 historical and 167 contemporary samples at 46 SNP loci revealed 82 unique multi-locus genotypes. The unique genotypes clustered into five groups and the majority of isolates collected in 2014 were genetically closely related, regardless of source location. The historical samples, representing several races, showed greater genetic differentiation. Overall, the SNP data indicate much of the genotypic variation found within fields was produced during asexual development, whereas overall genetic diversity may be influenced by sexual recombination on broader geographical and temporal scales.

## Introduction

Spinach (*Spinacia oleracea*) is believed to be native to the Middle East and has been cultivated for more than 1,300 years [1,2]. It became established in Europe in the 11^th^ century and was introduced to the new world and southern hemisphere in the 16^th^ century [3].

Global demand for spinach has increased substantially during recent decades. In 1961, world production of spinach was 2.96 million tons. By 2012, production increased seven fold to 21.66 million tons worldwide. In the United States, spinach production increased from 200,000 tons in 1961 to 354,000 tons in 2012 [4]. Per capita consumption of fresh market spinach increased from 0.3 kg/person in 1995 to 1.0 kg/person by 2005 [5]. The increased demand can largely be attributed to the popularity of pre-packaged baby spinach leaves and the health benefits associated with spinach including vitamins C and A, lutein, iron, folic acid, and magnesium [6,7].

Increased demand for spinach has dramatically influenced production practices. In California, planted acreage has expanded rapidly during the last two decades, 12-month production cycles are now common in the state, accompanied with high-density plantings, and reduced use of rotation crops. Furthermore, spinach seed usage has changed from the use of approximately 40,000 seed per acre to 4.0 million seeds per acre; dramatically changing plant population densities (Correll, personal observation). As a result, the higher plant population densities have influenced disease pressure. Increased market demand has also affected the spinach seed production industry. Although some open pollinated lines of spinach are produced, hybrid seed represents over 90% of production. Hybrid spinach lines are produced by the use of separate male and female inbred lines with different resistance characteristics for downy mildew disease [8,9].

*Peronospora effusa* causes downy mildew disease in spinach, and is one of the most economically important diseases constraining spinach production [8]. It is an obligate, heterothallic pathogen with two mating types (P1 and P2) [10]. Initial symptoms of downy mildew on spinach include yellow, irregular and chlorotic lesions on leaves. Blue-gray sporangia form when conditions are wet or during periods of high humidity [2]. If conditions are dry, lesions can desiccate and turn white or tan [11]. Sporangia are dispersed by wind, rain, and splashed irrigation water, and cause secondary cycles of disease development [2].

Isolates of *P*. *effusa* can be classified into distinct races based on infection phenotypes on a standardized set of different spinach cultivars [12]. The rate of classification of new races of *P*. *effusa* has exponentially increased in recent years with 15 races described [13]. The first race of *P*. *effusa* was identified in 1824, and in 1950, resistance to race 1 was determined to be conveyed by a single dominant gene. Race 2 was described in 1958 and soon thereafter resistance to both race 1 and race 2 was discovered [8]. While resistance to race 1 and 2 was initially thought to be controlled by a single gene, resistance against races 1 and 2 was later demonstrated to be controlled by two closely linked genes [14]. In 1976, race 3 was identified and resistance to it was incorporated into commercial hybrids two years later [8]. Race 4 was identified in 1990 in the United States [8,15] and resistant hybrids were introduced soon after [1]. Since 1990, 11 new races of *P*. *effusa* have been identified [5,12,13]. In 2000, a standardized set of race differentials for spinach was established by the International Seed Federation (ISF) to address confusion regarding races 5 and 6 in United States and Europe [16]. Currently, the International Working Group on Peronospora (IWGP) meets annually to evaluate emergence of novel, or deviating isolates, and assess their potential importance to global spinach production. Multiple laboratories run parallel tests and, if the results consistently indicate unique virulence, a new race number is assigned [13].The exact cause for the rapid emergence of new races is unknown, but is postulated to result from intense selective pressure associated with new spinach production practices [8].

Management of spinach downy mildew is problematic. Cultural controls include crop rotation, reduction of “green bridges”, and efforts to reduce free moisture on leaves. Although there are some fungicides available for management of spinach in conventionally grown production fields, approximately 40% of spinach grown in California and Arizona is produced organically where synthetic fungicides are prohibited. Thus, disease resistance is the most economical and environmentally sound means of managing downy mildew in both conventional and organically produced spinach.

Oospores have been detected in washings of seeds and infected seedlings were produced from contaminated seed [10]. Oospores associated with infected seeds can potentially survive for two years [9]. Although not common, oospores of the spinach downy mildew pathogen were detected in infected spinach tissue in California in 2007 [6]. In addition, oospores have been recovered from seed washes and strong circumstantial evidence indicates that new races of the pathogen have been introduced into areas where spinach had not been previously grown (Correll et al, unpublished). The relationship between detection levels and the role of seedborne inoculum in field epidemics is currently unknown [6]. A multiplex real-time PCR assay was recently created to detect various pathogens associated with spinach seeds, including *P*. *effusa*. When used to assess seed lots, a high percentage of seeds tested positive for contamination via PCR, but infected seedlings were not observed in grow-out studies (6). This discrepancy may reflect the presence of inviable propagules of the pathogen, lack of seed transmission among certain isolates, or inadequate conditions for seed transmission in the experimental conditions.

To better understand the emergence of new races of *P*. *effusa*, assessments of population diversity on various spatial and temporal scales are critical. For example, new races could originate via spontaneous mutation among prevalent genotypes, or could originate from selective sweeps due to the introduction of new genotypes on infected plant material. Initial population genetics studies in *P*. *effusa* were based on assessments of rDNA sequences and suggested low levels of genetic diversity among isolates [3,6]. However, subsequent analyses based on mitochondrial DNA allowed isolates to be classified by geographical origin (Group I for Asian and Oceania and Group II for American, European, and two Japanese samples). All base substitutions in the two mitochondrial genes tested were transitions, suggesting a recent genetic divergence. Because *P*. *effusa* is an obligate pathogen, the disease is postulated to have spread with the crop and is thought to have originated in a small geographic area [17]. However, the limited amount of genomic resources for *P*. *effusa* has hindered efforts to utilize more sophisticated molecular markers to assess genetic diversity in the context of evolutionary biology.

Our goal was to develop novel single nucleotide polymorphism (SNP) markers for *P*. *effusa* and to characterize diversity in a historical panel of race types and contemporary field populations in Arizona and California.

## Materials and Methods

### Sample collection and genomic DNA extraction

All samples were collected from commercial production fields of spinach and in each case, at every location; the grower (owner of the crop) gave permission to collect infected plant tissue. The specific geographic coordinates for each of the fields is not included as the growers requested this information remain private. Infected leaves were collected from spinach production sites in Yuma, Arizona in March of 2014 and from the Salinas Valley in May of 2014 and stored for 3 to 7 days on ice or in a refrigerator at 4°C. A disc (approximately 7mm diameter) of infected tissue was excised from each leaf and processed for genomic DNA extraction in a 96-well plate as previously described [18]. Briefly, this involves freeze drying the tissue in a 2ml deep-well plate followed by disruption into a powder and subsequent genomic DNA extraction using a silica-based approach.

The historical race-type panel of isolates is maintained at the University of Arkansas (Table 1). The races have been previously characterized and maintained as references for phenotypic assays. Sporangia are routinely produced on susceptible plants grown in a growth chamber inoculation assay and washed off and freeze dried prior to DNA extraction as outlined above.

**Table 1:** Historical panel of isolates and race types (if known or newly proposed).

Name	Race
UA1014APLP	Proposed 15
UA2509A	10
INT-1	10
UA2411	13
UA1411A	13
UA2012	10
UA1512A	Unknown
UA1812B	14
UA1412	14
UA0811	13
UA508B	4
UA2111	13
UA2012	10
UA2112	3
UA2412	13
UA2111	13
UA1410	11
UA4410	14
UA1210C	Unknown
UA2708PL	11
UA2511A	12+13
UA510C	13
UA3111	14
UA2209	12
1812A	14
A1013	13

### Candidate SNP identification

Genomic DNA was extracted from two isolates of *P*. *effusa*, UA2209 and UA4410 (race 12 and 14 respectively) and sequenced using 100bp paired-end Illumina sequencing on a HiSeq 2500 device (BGI, China). The sequencing is part of a larger effort to develop a reference genome for *P*. *effusa* (underway and to be reported separately). Here the sequence data was exclusively used to identify putative SNP sites. Raw sequence data (≈145M paired reads per isolate) was assembled *de novo* into contigs using CLC Genomics Workbench 7.5 (www.clcbio.com, Qiagen, Arhus) at default settings. All further sequence manipulation, mapping and genotyping were accomplished with CLC. The resulting 300 largest contigs for isolate UA2209 were processed further to identify all open reading frames (ORFs) greater than 1000 amino acids (3000bp). Of these, the largest 100 contigs with ORFs showing high predicted protein similarity, as assessed using Blastx in CLC, to other oomycete organisms (e.g. *P*. *infestans*) were used as a reference. The raw data from isolates UA4410 and UA2209 was then mapped separately to the 100 contig reference using settings of 90% similarity and 90% sequence coverage to identify heterozygous positions. Variable positions were assigned when the alternate allele frequency was >15% and <85%. Polymorphic SNP sites that fell within the 1000 amino acid putative genes and are predicted to be synonymous (non-amino acid changing) were then catalogued and used to choose 72 SNPs residing on separate contigs for further analyses.

### Targeted sequencing and genotype assignment

Individual primer pairs were designed using Batch Primer 3 (http://probes.pw.usda.gov/batchprimer3/) at default settings for generic primers with total amplicon size set as an optimum of 100bp with the amplified region containing the target SNP (S1 Table). The primer sequences and genomic DNA were submitted to Floodlight Genomics (FG, Knoxville, TN) for targeted sequencing. Floodlight Genomics uses a Hi-Plex approach to amplify targets (80-100bp) in a multiplexed PCR reaction and then generates sample specific sequences using a next-generation sequencing device (e.g. Ion Proton or Illumina MiSeq). The targeted sequencing was done at cost as part of the FG Educational and Research Outreach Program and the final data was obtained using an Ion Proton (Life Technologies). Binned raw sequence data obtained from FG was then used to map the sample-specific sequences to a reduced representation reference genome containing only the target sequences. SNP genotypes were assigned to target sites with at least 10X sequence coverage. Sites with <15% alternate allele frequency were considered homozygous genotypes.

### Genotype reproducibility

To confirm the reproducibility of the SNP genotypes, technical replications for amplifications and sequencing were conducted on a set of 96 samples that were processed separately on an Ion Torrent device, a MiSeq device and an Ion Proton device. In addition, 12 SNP positions previously genotyped using Sanger sequencing were included as positive controls.

### Genetic analyses

Population structure was assessed using Bayesian Markov chain Monte Carlo (MCMC) clustering model using the program STRUCTURE v2.3.4 [19,20]. The admixture model using sampling locations as prior was used with a burn-in of 2x10^5^ and 2x10^5^ iterations. The delta K was calculated and determined using Structure Harvester [21]. There were 10 independent runs for each K value from 1 to 30. GenAlEx was used to conduct a principle coordinate analysis of the clusters [22–24].

For each SNP locus the sample size, number of observations, availability, genotype number, allele number, major allele frequency, heterozygosity, gene diversity, and polymorphism information content (PIC) were calculated using the PowerMarker V3.25 software [25]. The number of observations is the number of non-missing DM genotypes. Availability is defined as 1−Obs / n, where Obs is the number of observations and n is the number of *P*. *effusa* isolates sampled. For each SNP locus, the number of genotype is 2 when no heterozygous individuals are observed or 3 when there are heterozygous individuals. Heterozygosity is the proportion of heterozygous individuals in the population; gene diversity is defined as the probability that two randomly chosen alleles from the population are different; and PIC is a closely related diversity measure [25]. The Shannon Index (I), F-Statistics (Fst), and gene flow were estimated using PopGene (available at http://www.ualberta.ca/~fyeh/popgene.html) [26].

## Results

### Genotypes

Genomic DNA from 375 samples, including 33 samples from a historical panel of race types, was submitted for targeted sequencing. Of these, 167 field samples and 26 of the historical race types amplified well enough to be included in further analysis; including 68 samples from Yuma, Arizona and 99 from the Salinas Valley, California. The 26 race types included races 3, 4, and 10–14. Forty seven markers had a minimum of 10X sequence coverage for all 193 samples. One marker was fixed for homozygosity and removed and the remaining 46 markers were used for subsequent analysis. The alternate allele coverage for most samples generally fell into a distribution around 50%, suggesting most samples are diploid (data not shown).

### Genotype reproducibility and variability in the field

The genotypes for the 12 previously sequenced SNP positions were replicated and showed identical genotypes using the Ion Torrent, Ion Proton and MiSeq sequencing devices. The 96 field samples, analyzed on all three sequencing platforms, produced identical multi-locus genotypes for all 46 SNPs included in the study.

In total, there were 82 unique multi-locus genotypes. Of these, 21 genotypes were found more than once and assigned a genotype identifier of G1-G21 (Table 2). The alternate allele frequency in the collection of 82 unique genotypes ranged from 1 to 49% and averaged 27%, with the majority (74%) of the loci having alternate allele frequencies >20% (S1 Table). Genotype G1 was recovered from 27 samples (14% of the total analyzed samples) only in Yuma, Arizona on the organic and non-organically produced spinach cultivars Coati, Tasman, Carmel, Meerkat, and Dromedary. The second most common genotype, G2, was recovered exclusively from the Salinas Valley, California on the spinach cultivars Coati, Tasman, Carmel, Meerkat, Soloman, Platypus, Silverwhale, and PV1030. The third most frequent genotype, G3, was recovered from 17 samples from fields in Yuma and the Salinas Valley from Coati, Tasman, Silverwhale, Carmel, Banjo, Meerkat, and NH185. The G3 genotype also included a soon to be described new race (race 15) and is the only race-typed (or soon to be race-typed) sample that clustered with the majority of the field isolates.

**Table 2:** Summary data for identical genotypes of downy mildew with race-typed individuals in bold.

GenotypeIdentifier	# of samples	Location/Race	Spinach Varieties and race-typed individuals
G1	27	Yuma	Coati, Tasman, Carmel, Meerkat, Dromedary, Merlat
G2	21	Salinas	Soloman, PV1030, Tasman, Silverwhale, Carmel, Meerkat, Coati, Platypus
G3	17	Yuma/Salinas	**UA1014APLP**, NH185, Tasman, Silverwhale, Carmel, Banjo, Meerkat, Coati
G4	12	Salinas	PV1030, Tasman, Silverwhale, Carmel, Meerkat, Coati, Platypus
G5	8	Salinas	Soloman, Silverwhale, Meerkat, Coati, Platypus
G6	7	Yuma	Coati, Merlat
G7	6	Yuma/Salinas	NH185, Plover, Cello, Dromedary
G8	4	Yuma	Cello, Dromedary
G9	3	Yuma	Plover, Meerkat, Merlat
G10	3	Salinas	Soloman, Platypus, Silverwhale
G11	3	Yuma	Tasman
G12	3	Salinas	Silverwhale, Soloman, Coati
G13	2	10	**UA2509A, INT-1**
G14	2	13	**UA2411, UA1411A**
G15	2	Salinas	Calisto
G16	2	Yuma/Salinas	Gazelle, Dromedary
G17	2	Yuma	Molakai
G18	2	Salinas	Meerkat, Silverwhale
G19	2	Salinas	Silverwhale, Meerkat
G20	2	Salinas	Meerkat, Coati
G21	2	Salinas	Banjo, Meerkat

While most identical genotypes were recovered from field samples, there were two cases, G13 and G14, where identical genotypes were recovered for two race 10 (G13) and two race 13 (G14) samples (Table 2).

### Population structure

**Fig 1:**
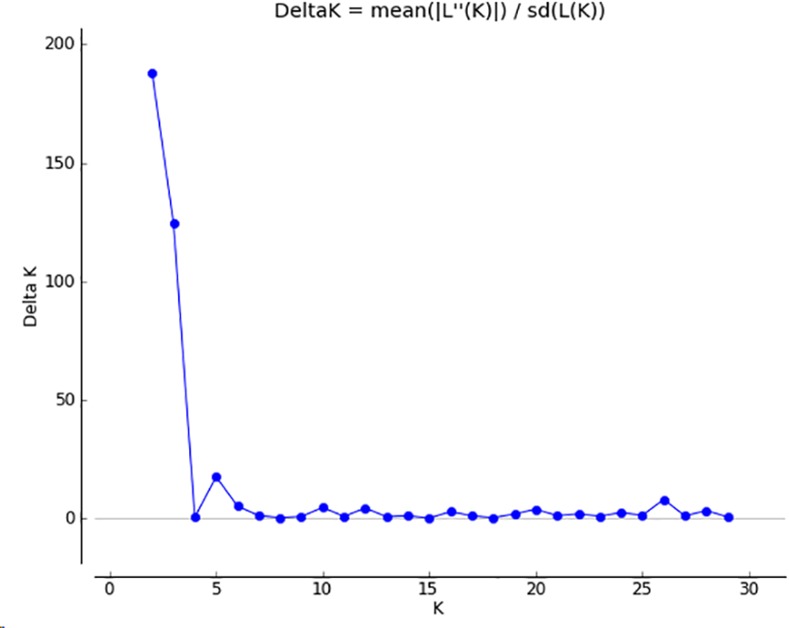
STRUCTURE HARVESTER results for downy mildew showing a K value of 5.

**Fig 2:**
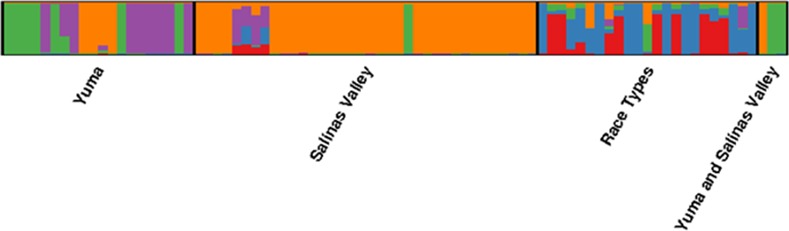
STRUCTURE plot of assigned probabilities of 82 unique genotypes. Each genotype is represented by a bar indicating likelihood of membership in cluster 1 (red), cluster 2 (blue), cluster 3 (green), cluster 4 (purple), and cluster 5 (orange).

**Fig 3:**
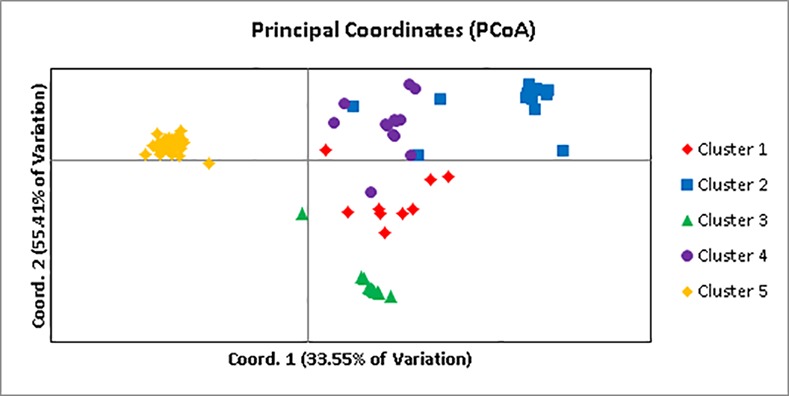
Principle Coordinates Analysis showing genetic distances between individuals in five clusters. Each color represents a STRUCTURE cluster. Cluster 1 (red), cluster 2 (blue), cluster 3 (green), cluster 4 (purple), and cluster 5 (orange).

Analysis using the program STRUCTURE indicated a K value of 5 based on the largest ln Pr(X|K) value (Fig 1). Fig 2 is a structure graph showing the genotypes divided by location and assigned a color based on cluster. The first cluster includes 10 genotypes from the panel of race types including races 3, 10–12, and 14. The second cluster includes 13 races including races 4 and 10–14. The third cluster has 11 unique genotypes and includes samples from Yuma and Salinas Valley as well as a representative of race 11. In the fourth cluster, 13 samples from both Yuma and Salinas Valley were collected from eight different spinach cultivars. The fifth cluster is the largest cluster with 34 unique genotypes and includes field samples from both Yuma and Salinas Valley, collected from 10 different spinach cultivars. A principle coordinate analysis supports the STRUCTURE groupings showing moderate to high levels of separation between the clusters (Fig 3).

All 46 SNPs assessed in the 82 unique *P*. *effusa* genotypes had no missing data (S2 Table). Most SNP loci (84.8%) had heterozygous individuals and the mean of heterozygosity was 0.37. The major allele frequency averaged 0.73 with a range between 0.51 and 0.99, indicating a wide range of allele frequencies. Both gene diversity and PIC also showed a wide range with 0.01–0.50 (averaged 0.36), and 0.01–0.37 (0.29), respectively, indicating there is variation among the 82 isolates. The Shannon Index (I), F-Statistics (Fst), and gene flow estimates from PopGene averaged 0.53, 0.21, and 0.96, respectively, and also showed wide ranges with 0.04–0.69, 0.0–0.51, and 0.24–83.78, respectively, indicating SNP variation and gene flow exists and there is genetic diversity among the 82 isolates. AMOVA analysis indicates the largest proportion of diversity (20.5%) is distributed among the five proposed Structure groups whereas between 3 and 9% of the total variation is contained within specific groups (S3 Table).

## Discussion

This is the first fine-scale assessment of population diversity for *P*. *effusa* using SNP markers. The inclusion of historical reference races was useful as it showed these markers have a reasonably high level of polymorphism suitable for population analyses.

Genotypes from field samples collected in Yuma and Salinas in 2014 mostly differ from the historical panel of race types. Identical genotypes can be found in both Yuma, Arizona and Salinas Valley, California and closely related genotypes from both locations consistently grouped together. Most samples in 2014 had very similar genotypes suggesting there is movement of inoculum between the two production regions during the concurrent growing seasons or there is a common source of downy mildew inoculum. Spinach material and equipment are moved between the two production areas and movement of infected plant material is highly plausible.

Isolate UA1014APLP, a newly identified isolate with a novel race profile (Feng and Correll, unpublished), was the only isolate from the race panel that was found in STRUCTURE group five, the largest group of field samples. This isolate is a new race and is currently in a ring test being conducted by the International Working Group on Peronospora to be nominated as a new race. Closely related genotypes to this isolate dominated the field populations in 2014. The appearance of this new race (and the many genotypes similar to it) is likely due to selection pressure imposed by growers who have increasingly relied on spinach cultivars resistant to races 1–15 in Yuma and the Salinas Valley.

Although some historical races cluster together (e.g. race 14) there is not a strict correlation between genotype and race type. Therefore, it is not possible with this panel of neutral SNP markers to strictly assign a race type based on multi-locus genotype. However, as more markers become available, and more information is generated on the genome of this pathogen, progress can be made toward this goal.

It is becoming increasingly clear that oomycete plant pathogens have the potential to produce significant amounts of genotypic variation during asexual population expansion [27]. The majority of the genotypes recovered during the 2014 season are very similar and due to the number of heterozygous sites in common, it is likely the differences observed are due to asexual phenomenon, such as loss of heterozygosity. A clear example of loss of heterozygosity was found when comparing SNP positions between isolates UA2209 (race 12) and UA4410 (race 14). The race 14 isolate was detected approximately 1.5 years after the race 12 isolate [13]. Both isolates are highly heterozygous and the distribution of heterozygous sites was identical across 83 of the 100 contigs used for this work. The only difference was that isolate UA4410 shows a loss of heterozygosity (only one of the two possible haplotypes is present) across 17 of the 100 contigs relative to UA2209. A similar phenomena has been documented at the genome scale with the vegetable pathogen, *Phytophthora capsici*, and was found to be associated with changes in pathogenicity, virulence and mating type [28]. How this loss of heterozygosity occurs is unknown and the specifics of our findings with downy mildew will be presented in a genome-wide comparison study that is currently underway. It’s possible that the ability to tolerate loss of heterozygosity provides an important avenue for adaptive evolution in epidemic populations. More work is needed in this area.

Although the 2014 epidemics appear to be dominated by a few clonal lineages with sub-clonal variation, there appears to be sufficient genotypic diversity in the historical race panel to suggest sexual as well as asexual reproduction contributes to diversity over time. Sexual reproduction of the spinach downy mildew pathogen has been documented based on the observation of oospores in California [6] and in seed washes (Correll and Feng, unpublished). Further temporal studies to track genotypic diversity in parallel with the deployment of novel spinach cultivars will be helpful to better understand how this important pathogen is able to rapidly overcome novel resistant plant varieties and may be useful to determine the importance, overall, of sexual vs. asexual mechanisms for driving variation in epidemic populations.

The current study documents a relatively high level of genotypic diversity within a regional sample of the spinach downy mildew pathogen and indicates genomic variation within a clonal context is common. This initial study is a useful baseline to address how asexual plasticity may impact evolving virulence of the pathogen.

## Supporting Information

S1 Table Primers and allele frequencies. (XLSX)

S2 Table Basic population genetic statistics for *Peronospora effusa* isolates. (XLSX)

S3 Table Analysis of molecular variance (AMOVA). (XLSX)
